# Efficacy of progestin-primed ovarian stimulation in women with polycystic ovary syndrome undergoing *in vitro* fertilization: a systematic review and meta-analysis

**DOI:** 10.3389/fendo.2023.1224858

**Published:** 2023-09-19

**Authors:** Liu Yang, Fuxiang Liang, Yue Yuan, Xufei Luo, Qi Wang, Liang Yao, Xuehong Zhang

**Affiliations:** ^1^ The First Clinical Medical College, Lanzhou University, Lanzhou, China; ^2^ The First Hospital of Lanzhou University, Lanzhou, China; ^3^ Key Laboratory for Reproductive Medicine and Embryo of Gansu Province, The First Hospital of Lanzhou University, Lanzhou, China; ^4^ Department of Thoracic Surgery, The Second Affiliated Hospital, School of Medicine, Zhejiang University, Hangzhou, China; ^5^ Evidence-Based Medicine Center, School of Basic Medical Sciences, Lanzhou University, Lanzhou, China; ^6^ Department of Health Research Methods, Evidence, and Impact, McMaster University, Hamilton, ON, Canada

**Keywords:** progestin-primed ovarian stimulation, polycystic ovary syndrome, *in vitro* fertilization, intracytoplasmic sperm injection, meta-analysis progestin-primed ovarian stimulation, meta-analysis

## Abstract

Polycystic ovary syndrome (PCOS) is a common endocrinopathy causing infertility in childbearing women. Progestin-primed ovarian stimulation (PPOS) protocol has recently been used for infertile women. However, whether PPOS provides a significant benefit over gonadotropin-releasing hormone (GnRH) analogue protocols in PCOS is still controversial. The objective of this systematic review is to investigate the efficacy of PPOS in patients with PCOS during *in vitro* fertilization (IVF) or intracytoplasmic sperm injection (ICSI). We searched Medline, Embase, Google Scholar, ClinicalTrials, and Cochrane Central Register of Controlled Trials from inception to April 1, 2023. Randomized controlled trials (RCTs) and observational studies comparing the efficacy between PPOS and conventional GnRH analogue protocols in patients with PCOS in English were included. The primary outcomes included live birth rate, the incidence of moderate or severe ovarian hyperstimulation syndrome (OHSS), and the number of metaphase II oocytes. The pooled estimates were calculated using the random-effects models as odds ratios (OR) or mean differences (MD) with 95% confidence intervals (CIs). Three RCTs and six cohort studies involving 2289 patients were included. Results from RCTs suggest that PPOS leads to no significant difference in the risk of OHSS, the number of metaphase II oocytes, or the rate of live birth when compared to GnRH analogue protocols. The pooling estimates of cohort studies showed consistent results. Additionally, in cohort studies, PPOS required a higher dose of Gn and tended to improve the implantation rate, clinical pregnancy rate, and ongoing pregnancy rate. For subgroup analyses, the higher implantation rate, clinical pregnancy rate, and ongoing pregnancy rate were found in PPOS compared to the GnRH agonist short protocol. However, the certainty of the evidence for the outcomes was generally low. Overall, There is currently no evidence to support that PPOS could reduce the risk of OHSS, increase oocyte maturation, or improve pregnancy outcomes in women with PCOS undergoing IVF/ICSI when compared to GnRH analogue protocols. Considering its efficiency and safety, this protocol could be a patient-friendly and viable alternative for PCOS patients, especially when frozen-thawed embryo transfer is planned. Future high-quality randomized trials with children’s long-term safety and cost-effective analyses are still required.

**System Review Registration:**

NPLASY (202340059). https://inplasy.com/inplasy-2023-4-0059/

## Introduction

Polycystic ovary syndrome (PCOS) is a common endocrine disorder that can cause infertility in women of childbearing age. Approximately 80% of infertility cases involving ovulatory dysfunction are related to PCOS (1). According to current recommendations, *in vitro* fertilization/intracytoplasmic sperm injection (IVF/ICSI) can be considered a third-line treatment option for women with PCOS after other ovulation induction methods have failed. However, unlike other etiology of infertility, patients with PCOS have a unique reproductive and metabolic milieu characterized by hyperandrogenism, insulin resistance, and a strong ovarian response to gonadotropin stimulation, which can lead to poor-quality oocytes, high rates of early miscarriage, and an increased risk of ovarian hyperstimulation syndrome (OHSS) (2, 3). Therefore, individualized controlled ovarian stimulation (COS) treatment is necessary for these patients.

Current guidelines recommend gonadotropin-releasing hormone (GnRH) antagonist protocols as the primary COS protocol for PCOS patients (4). This is because it reduces the duration of stimulation, total gonadotrophin dose, and incidence of OHSS compared to traditional GnRH agonist protocols. However, the GnRH antagonist protocol may reduce the number of oocytes retrieved (5, 6) and increase cycle cancellation rates (7).

Previous studies have reported that progesterone can prevent moderate to severe OHSS in COS cycles (8). As a result, a new progesterone protocol, progestin-primed ovarian stimulation (PPOS), has been gradually applied to COS cycles since 2015 (9). This protocol is based on theories that high progesterone levels can affect the frequency of GnRH pulses, inhibit premature luteinizing hormone (LH) surges, and suppress pituitary function (7). So far, PPOS has been successfully used in patients with normal ovarian response (9), low response (10), PCOS (11), and endometriosis (12). However, as early exposure to high levels of progesterone can change endometrial receptivity and lead to asynchronous development between the embryos and endometrium (13), the “freeze all” strategy - where all embryos are cryopreserved without fresh embryo transfer - is required for the protocol. Fortunately, advances in vitrification have made it possible to reliably and reproducibly freeze and thaw embryos for preservation and transplantation (14). In addition, oral progestins are less expensive (15) and do not require injection (13) compared to GnRH analogues, which can improve patients’ compliance. Therefore, the PPOS protocol is recognized as a viable option for PCOS patients.

However, the benefits of the PPOS protocol for patients with PCOS-related infertility are still controversial. For example, a previous randomized trial showed that this new progesterone protocol did not improve the cumulative pregnancy rate or reduce the risk of moderate/severe OHSS for women with PCOS (16), while a recent cohort study (17) suggested it was associated with a higher implantation rate, clinical pregnancy rate, and live birth rate. Thus, conducting a systematic review and meta-analysis is necessary to provide evidence clarifying the efficacy of the PPOS protocol for infertile women with PCOS undergoing IVF/ICSI.

## Materials and methods

### Protocol and registration

This systematic review was conducted following the Cochrane Handbook for systematic reviews of interventions (18) and registered in the International Platform of Registered Systematic Review and Meta-Analysis Protocols (INPLASY) with the number INPLASY 202340059. We also followed the Preferred Reporting Items for Systematic Reviews and Meta-Analyses (PRIMSA) checklist to report this study (**Supplementary Table 1**) (19).

### Ethics

As this study was a systematic review and meta-analysis including previously published data, institutional review board approval was not required.

### Search strategy

We searched Medline (via PubMed), Embase, Google Scholar, ClinicalTrials, and Cochrane Central Register of Controlled Trials (CENTRAL) from inception to April 1, 2023, without any limitation by publication status or sample size. Additionally, we manually checked conference proceedings’ references and identified studies or websites of the clinical trial registry to obtain additional relevant data. The search terms used in PubMed are listed in **Supplementary Table 2**.

### Study selection

Studies meeting the following criteria were included: 1) RCTs or observational studies published in English; 2) infertile women diagnosed with PCOS undergoing IVF or ICSI; 3) the intervention group used PPOS protocol without discrimination of progestin types, and the control group included GnRH analogue protocols, involving the GnRH antagonist and the GnRH agonist (GnRH-a) protocol; and 4) primary outcomes included: live birth rate; the incidence of moderate or severe OHSS; and the number of metaphase II (MII) oocytes; Secondary outcomes included the number of oocytes retrieved; the number of good-quality embryos; the total dose of gonadotropin (Gn) stimulation; the incidence of premature LH surge; cycle cancellation rate (due to no viable embryos); implantation rate (IR); clinical pregnancy rate (CPR); and ongoing pregnancy rate (OPR).

The titles and abstracts of the records were independently reviewed by two authors (L.Y. and Y.Y.), and then the full texts considered potentially relevant were screened. Any disagreements were resolved through discussion with a third reviewer (F.X.L.).

### Data extraction

Two authors (L.Y. and F.X.L.) independently extracted the data and cross-checked their findings. Any discrepancies were discussed with a third author (X.H.Z.). We pulled the following information: first author, country, publication year, study design, inclusion criteria, exclusion criteria, number of patients, female age, details of COS protocols, and primary outcomes as reported.

### Risk of bias and certainty of evidence assessment

Potential methodology bias in each included study was independently assessed by two authors (Q.W. and X.F.L.). Any differences were resolved through discussion with a third author (X.H.Z.). We evaluated the risk of bias in RCTs and observational studies with the Cochrane Risk-of-Bias tool (20) and Newcastle-Ottawa Scale (NOS) (21), respectively. Each part was graded as “low risk,” “unclear risk,” or “high risk.”

We independently assessed the certainty of the evidence for each outcome according to the Grading of Recommendation, Assessment, Development and Evaluation (GRADE) system (22–24), and we reported the grading results following the principles of GRADE guidance (23).

### Data synthesis

Statistical analysis was performed by Review Manager program version (RevMan) 5.4 (Cochrane Collaboration, Oxford, UK). Due to the inevitable clinical and potential heterogeneity, a random-effects model was chosen to perform meta-analysis (25). For dichotomous outcomes, we calculated the odds ratio (OR) with 95% confidence intervals (CIs) (25). For continuous data, we pooled the results for meta-analysis as the mean difference (MD) with 95% CIs. The I-squared (*I^2^*) was applied to reflect the heterogeneity, with substantial heterogeneity considered to exist when *I^2^* > 50% (26, 27).

We also performed subgroup analysis based on predefined factors to explain potential sources of heterogeneity between studies (26). The predefined factors included the control group’s COS protocols (GnRH antagonist or agonist protocols) and the types of oral progestins (Medroxyprogesterone acetate, Utrogestan, or Dydrogesterone) in PPOS.

Furthermore, funnel plots were used to investigate potential publication bias when more than nine studies were included in the meta-analysis (28).

## Results

### Study selection

**Figure 1:**
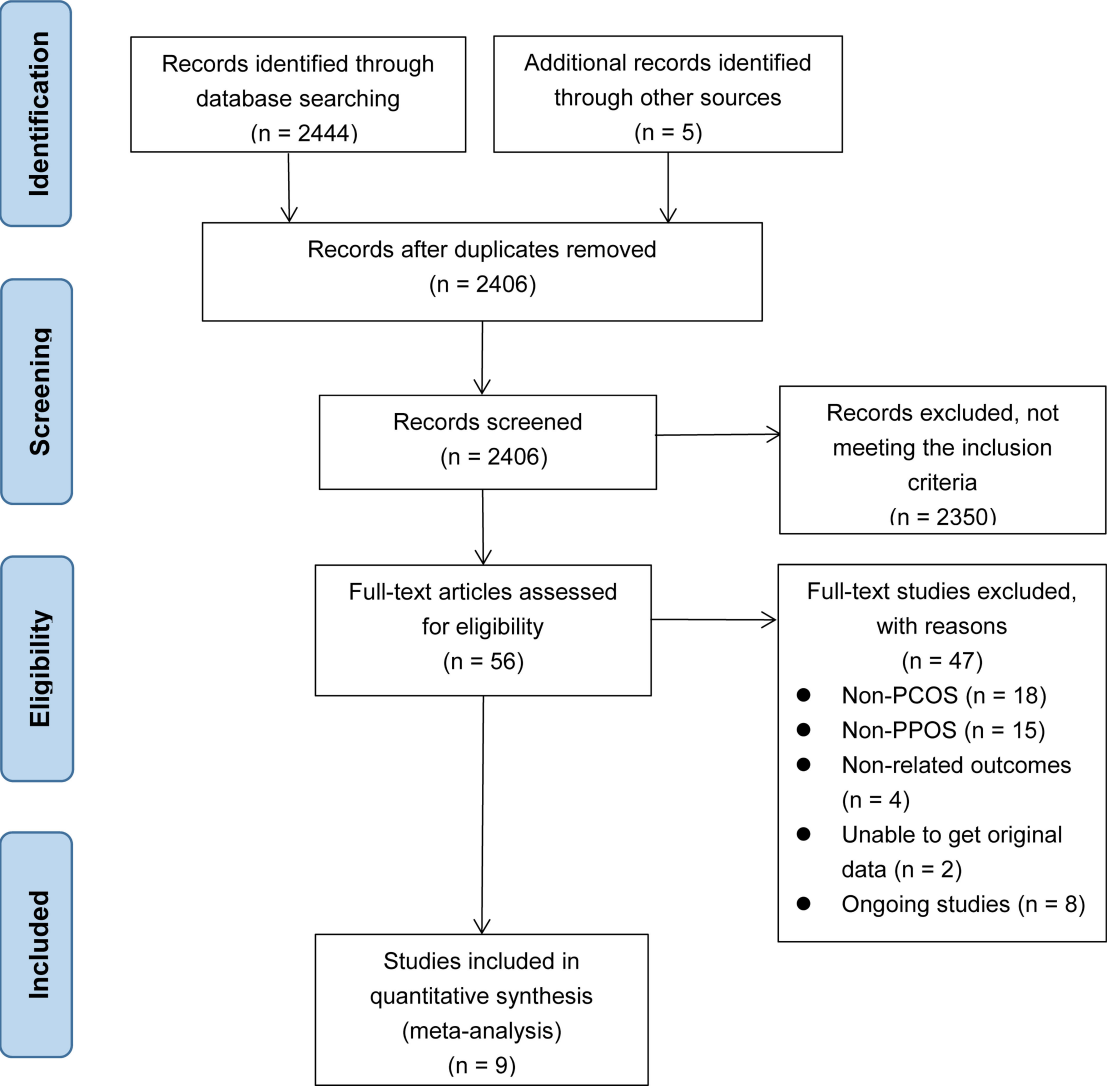
Flow chart of study selection for the systematic review and meta-analysis.

Initially, we identified 2444 records by searching the database, and an additional five records were obtained from other sources. After removing 43 duplicate records, we included 2406 records. Among them, 2350 records were excluded based on title and abstract. After reading the full texts of the remaining 56 studies, 47 were excluded: 18 were excluded for non-PCOS, 15 for irrelevant intervention measures, four for non-relevant outcomes, eight for ongoing studies, and the remaining two for missing original data. Finally, nine studies (11, 15–17, 29–33) were included in our study. The literature search and study selection process is shown in **Figure 1**.

### Characteristics of the included studies

We included three RCTs (15, 16, 33) and six retrospective cohort studies (11, 17, 29–32) involving 2289 women. Most included studies were conducted in China (11, 15, 17, 30–33), and the others were in Japan (29) and Iran (16). The average age of women was 28-34 years old. Four studies (15, 17, 31, 32) applied medroxyprogesterone acetate (MPA) as the oral progestin in the PPOS group; two used GnRH-a short protocol in the control group, and the other two used GnRH antagonist protocol. Three studies (16, 29, 30) used dydrogesterone in the PPOS group, with GnRH antagonist protocol in all control groups. The remaining two studies (11, 33) used Utrogestan as the oral progestin, either GnRH-a short protocol or GnRH antagonist protocol in the control group. The specific information of included studies is presented in **Table 1**.

**Table 1:** Characteristics of studies included in the meta-analysis.

Study, year	Country	Study design	Inclusion criteria	Exclusion criteria	PPOS group		Control group		Primary Outcomes
					No. of patients Age (year)	COS protocol	No. of patients Age (year)	COS protocol	
Wang et al., 2016 (15)	China	RCT	(1) Female aged 18-39 years old (2) Rotterdam criteria of PCOS diagnosis	(1) Clinically significant systemic disease (2) Endometriosis grade 3 or higher (3) Documented ovarian failure (4) Received hormonal treatments in the previous three months	60 30.35 ± 3.10	HMG + MPA 10 mg/day Trigger: GnRHa 0.1 mg + hCG 1000 IU	60 29.88 ± 3.14	Short Protocol HMG + Decapeptyl 0.1 mg Trigger: hCG 2000	OHSS, OPR
Eftekhar et al. 2019 (16)	Iran	RCT	(1) Female aged 18-40 years old (2) Rotterdam criteria of PCOS diagnosis	(1) Previous intrauterine abnormalities (submucosal fibroma, uterine polyp, and intrauterine adhesions) (2) Severe endometriosis (3) Systemic diseases (4) Azoospermia in male partners	58 28.47 ± 3.60	Cinnal-f + Dydrogesterone 20 mg/day Trigger: decapeptyl 0.2 mg + hCG 1000 IU	60 28.98 ± 3.55	GnRH antagonist Protocol Cinnal-f + cetrorelix 0.25 mg Trigger: decapeptyl 0.2 mg + hCG 1000 IU	CPR
Zhu et al. 2021 (33)	China	RCT	(1) Female aged 18-38 years old (2) Rotterdam criteria of PCOS diagnosis (3) BMI < 28 kg/m2	(1) Previous IVF/ICSI history (2) Severe endometriosis (3) Significant systemic disease (4) Unsuitable for ovarian stimulation (5) Receipt of hormonal drugs in the past three months	60 29.65 ± 3.16	HMG + Utrogestan 100 mg/day Trigger: decapeptyl 0.1 mg	60 28.80 ± 2.84	GnRH antagonist Protocol HMG+ cetrorelix 0.25 mg Trigger: decapeptyl 0.1 mg	No. of oocytes retrieved
Xiao et al. 2019 (32)	China	Retrospective cohort study	(1) Female aged 20-40 years old (2) Rotterdam criteria of PCOS diagnosis	Other endocrine dysfunction or indications for IVF	67 32.31 ± 4.62	HMG + MPA 10 mg/day Trigger: decapeptyl 0.2 mg + hCG 2000 IU	90 30.69 ± 3.99	GnRH antagonist Protocol Recombinant FSH + cetrorelix 0.25 mg Trigger: decapeptyl 0.2 mg + hCG 2000 IU	OHSS, CPR
Zhu et al. 2016 (11)	China	Retrospective cohort study	(1) Female aged 18-38 years old (2) Rotterdam criteria of PCOS diagnosis	(1) Endometriosis grade 3 or higher (2) Documented ovarian failure (3) Previous cycles with no oocyte retrieved	123 29.15 ± 2.94	HMG + Utrogestan 200 mg/day Trigger: triptorelin 0.1 mg	77 29.77 ± 3.12	Short Protocol HMG + triptorelin 0.1 mg Trigger: hCG 3000IU	Viable embryo rate
Gurbuz et al. 2020 (26)	Turkey	Retrospective cohort study	(1) Female aged 20-39 years old (2) Rotterdam criteria of PCOS diagnosis (3) Body weight > 50 kg	(1) Severe endometriosis (2) Severe male factors (3) Unsuitable for ovarian stimulation (4) Uterine or ovarian abnormalities, or endocrinological abnormalities	258 29.08 ± 4.40	Recombinant FSH + Dydrogesterone 20 mg/day Trigger: triptorelin 0.2 mg	267 29.24 ± 4.00	GnRH antagonist Protocol Recombinant FSH + cetrorelix 0.25 mg Trigger: triptorelin 0.2 mg	Incidence of premature LH surge
Huang et al. 2021 (30)	China	Retrospective cohort study	(1) First IVF/ICSI treatment (2) Rotterdam criteria of PCOS diagnosis (3) Body weight between 50-70 kg	(1) Female aged > 38 years (2) Basal FSH level > 12 mIU/mL (3) Previous ovarian surgery, congenital uterine anomaly, or intrauterine adhesion (4) Male partner with non-obstructive azoospermia	173 34.2 ± 2.8	Corifollitropin-alfa/Recombinant FSH + Dydrogesterone 20 mg/day Trigger: leuprolide acetate 1 mg	160 34.4 ± 2.6	GnRH antagonist Protocol Corifollitropin-alfa/Recombinant FSH + cetrorelix 0.25 mg Trigger: leuprolide acetate 1 mg	Incidence of premature LH surge
Chen et al., 2021 (17)	China	Retrospective cohort study	(1) Female aged 20-40 years old (2) Rotterdam consensus of PCOS diagnosis	(1) Fresh transfer cycles (2) Female aged >40 years (3) Basal FSH level ≥10 IU/L (4) Endometriosis grade 3 or higher; history of ovarian surgery; uterine anomalies (5) History of recurrent spontaneous abortion (6) Abnormal chromosomal karyotype (7) Fetal reduction in the first FETcycles (8) Lost to follow-up	304 31.09 ± 3.41	HMG + MPA 10 mg/day Trigger: triptorelin 0.1 mg + hCG 1000 IU (n=243) or triptorelin 0.1 mg (n=42) or hCG 2000-3000IU (n=19)	152 31.18 ± 3.41	Short Protocol HMG + triptorelin 0.1 mg Trigger: hCG 2000-3000IU	IR
Li et al., 2020 (34)	China	Retrospective cohort study	(1) First IVF/ICSI treatment (2) PCOS diagnosis	NA	65 NA	NA	195 NA	GnRH antagonist Protocol Trigger: NA	No. of oocytes retrieved

BMI, body mass index; COS, controlled ovarian stimulation; CPR, clinical pregnancy rate; FET, frozen-thawed embryo transfer; FSH, follicle-stimulating hormone; GnRH, gonadotropin-releasing hormone; hCG, human chorionic gonadotropin; HMG, human menopausal gonadotropin; ICSI, intracytoplasmic sperm injection; IVF, in vitro fertilization; LH, luteinizing hormone; IR, implantation rate; LBR, live birth rate; MPA, medroxyprogesterone acetate; NA, not available; OHSS, ovarian hyperstimulation syndrome; OPR, ongoing pregnancy rate; PCOS, polycystic ovary syndrome; PPOS, progestin-primed ovarian stimulation; RCT, randomized controlled trial.

### Risk of bias

The risk of bias was performed in the risk of bias summary and NOS score (**Supplementary Figure 1**; **Supplementary Table 3**). For selection bias in RCTs, three trials (15, 16, 33) were judged at a low risk of random sequence generation, and two (15, 16) at a low risk of allocation concealment, as detailed methods were provided. For performance bias, one trial (15) was evaluated as having a low risk because of the strict double-blinding design, one (33) mentioned that no blinding of participants was judged at high risk, and the remaining trial was considered at unclear risk. Two studies (15, 33) presented explicit explanations about the blinding of outcome assessors, resulting in a low risk of detection bias. On the other hand, one study (15) didn’t describe the reason for the loss to follow-up, so it was assessed as having a high risk of attrition bias. All trials were free from reporting bias and other biases. For retrospective cohort studies, the NOS score ranged from 6 to 7. Unscored items generally were the adequacy of follow-up and whether the outcome was present at the start of the study.

### Meta-analysis of primary outcomes

**Figure 2:**
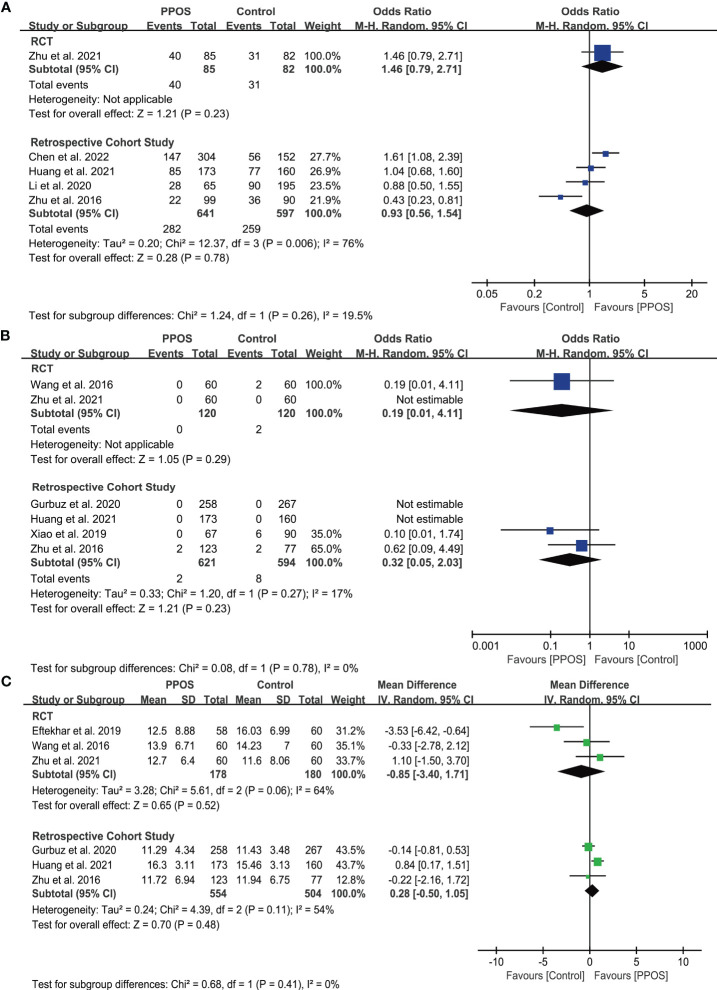
Forest plots of primary outcomes in infertile patients with PCOS. Progestin-primed ovarian stimulation (PPOS) versus gonadotropin-releasing hormone (GnRH) analogue protocols: **(A)** live birth rate (LBR), **(B)** incidence of ovarian hyperstimulation syndrome (OHSS), and **(C)** number of metaphase II (MII) oocytes.

Five studies, including one RCT (33) and four cohort studies (11, 17, 30, 31), reported the live birth rate (LBR) in women with PCOS. The pooled results from the cohort studies showed no significant difference between PPOS and GnRH analogue protocols in terms of LBR (OR = 0.93, 95% CI: 0.56-1.54, *I^2^ =* 76%, 1238 cycles), which was consistent with the RCT (OR = 1.46, 95% CI: 0.79-2.71, 167 cycles) (**Figure 2**). Only 2 (0.3%) and 10 (1.4%) women in the PPOS and GnRH analogue protocol groups, respectively, experienced moderate or severe OHSS. Pooled outcomes from RCTs (15, 33) (OR = 0.19, 95% CI: 0.01-4.11, two studies of 240 patients) and cohort studies (11, 29, 30, 32) (OR = 0.32, 95% CI: 0.05-2.03, *I^2^ =* 17%, four studies of 1215 patients) both showed no significant difference of the risk of OHSS between the two groups (**Figure 2**). Additionally, pooled analyses from RCTs (15, 16, 33) (MD = -0.85, 95% CI: -3.40-1.71, *I^2^ =* 64%, three studies of 358 patients) and cohort studies (11, 29, 30) (MD = 0.28, 95% CI: -0.50-1.05, *I^2^ =* 54%, three studies of 1058 patients) both found that PPOS and GnRH analogue protocols obtained a similar number of MII oocytes (**Figure 2**). However, the certainty of the evidence for primary outcomes was low (**Table 2**).

**Table 2:** The certainty of the evidence for primary outcomes.

Outcomes	Study design	Anticipated absolute effects* (95% CI)			Relative effect (95% CI)	No. of cycles (studies)	Quality of the evidence (GRADE)
		Risk with control	Risk with PPOS	Difference			
**Live birth**	RCT	378 per 1000	552 per 1000 (299 to 1024)	174 (79 to 646)	**OR** 1.46 (0.79 to 2.71)	167 cycles (1)	**Low^1^**
	Cohort	434 per 1000	404 per 1,000 (243 to 668)	-30 (-191 to 234)	**OR** 0.93 (0.56 to 1.54)	1238 cycles (4)	**Low**
**OHSS**	RCT	17 per 1000	3 per 1000 (0 to 70)	-14 (-17 to 53)	**OR** 0.19 (0.01 to 4.11)	240 patients (2)	**Low^2^**
	Cohort	13 per 1000	4 per 1000 (1 to 26)	-9 (-12 to 13)	**OR** 0.32 (0.05 to 2.03)	1215 patients (4)	**Low**
**MII oocytes**	RCT	The mean number of MII oocytes in the control group was 13.95.	The mean number of MII oocytes in the intervention group was 0.85 lower (3.40 lower to 1.71 higher).	-0.85 (-3.40 to 1.71)	NA	358 patients (3)	**Low^1^**
	Cohort	The mean number of MII oocytes in the control group was 12.79.	The mean number of MII oocytes in the intervention group was 0.28 higher (0.50 lower to 1.05 higher).	0.28 (-0.50 to 1.05)	NA	1058 patients (3)	**Low**

*The risk in the intervention group (and the 95% confidence interval) is based on the risk in the control group and the relative effect of the intervention.

CI, confidence interval; GRADE, Grading of Recommendation, Assessment, Development, and Evaluation; MII, metaphase two; NA, not available; OHSS, ovarian hyperstimulation syndrome; OR, odds ratio; PPOS, progestin-primed ovarian stimulation; RCT, randomized controlled trial.

^1^Downgraded two steps due to imprecision of results as shown in wide confidence intervals and heterogeneity.

^2^Downgraded two steps due to imprecision of results as shown in wide confidence intervals and the small number of events.

### Meta-analysis of secondary outcomes

Nine studies reported retrieved oocytes, and five reported good-quality embryo data for laboratory parameters. Regardless of whether the studies were cohort studies (11, 17, 29–32) (MD = -0.27, 95% CI: -0.98-0.43, *I^2^ =* 58%, six studies of 1,931 patients) or RCTs (15, 16, 33) (MD = -0.45, 95% CI: -3.11-2.20, *I^2 =^* 57%, three studies of 358 patients), there was no significant difference in the number of oocytes retrieved between the two groups. Similarly, there was no difference in the number of good-quality embryos between the groups, according to both cohort studies (11, 30–32) (MD = -0.01, 95% CI: -0.35-0.33, *I^2^ =* 24%, four studies of 950 patients) and the RCT (15) (MD = -0.02, 95% CI: -1.45-1.41, one study of 120 patients) (**Supplementary Figure 2**).

In terms of cycle characteristics, cohort studies (11, 17, 29, 32) have shown that the PPOS group had a significantly higher amount of Gn (MD = 234.31, 95% CI: 174.59-294.02 IU, *I^2^* = 0%, four studies of 1338 patients). Similarly, the PPOS group in RCTs (15, 16, 33) showed an increasing trend of Gn dose (MD = 179.57, 95% CI: -235.39-594.52 IU, *I^2^* = 94%, three studies of 358 patients), although the difference was not statistically significant. Moreover, there was no significant difference found in cycle cancellation rate between the groups, neither in RCTs (15, 16, 33) (OR = 0.65, 95% CI: 0.08-5.20, *I^2^* = 62%, three studies of 358 patients) nor in cohort studies (11, 17, 30, 32) (OR = 0.82, 95% CI: 0.43-1.56, *I^2 =^* 0%, four studies of 1146 patients) (**Supplementary Figure 3**). Additionally, four studies (15, 29, 30, 33) reported no cases of premature LH surge in any group.

Regarding fertility and pregnancy outcomes, the PPOS protocol demonstrated an increasing implantation rate (IR) trend compared to the control group. This was observed in both cohort studies (11, 17, 29, 30, 32) (OR = 1.29, 95% CI: 0.95-1.74, *I^2^* = 70%, five studies of 2694 embryos) and RCTs (15, 33) (OR = 1.17, 95% CI: 0.85-1.60, *I^2^* = 0%, two studies of 631 embryos). Additionally, nine studies reported clinical pregnancy rate (CPR) and ongoing pregnancy rate (OPR). From cohort studies (11, 17, 29–32), there was an increasing trend in CPR for PPOS (OR = 1.27, 95% CI: 0.98-1.65, *I^2^* = 39%, six studies of 1756 cycles), but this trend was not observed in RCTs (15, 16, 33) (OR = 1.00, 95% CI: 0.51-1.94, *I^2^* = 58%, three studies of 418 cycles). Similar results were found for the OPR (**Supplementary Figure 4**). However, the certainty of the evidence for secondary outcomes was generally low (**Supplementary Table 4**).

### Subgroup analysis

Subgroup analyses based on oral progestins showed that MPA and dydrogesterone required a higher Gn dose than the GnRH analogue group. However, no difference was found for utrogestan. Additionally, the PPOS group had higher IR, CPR, and OPR than the GnRH agonist short protocol group, but there was no difference in these outcomes between the PPOS group and the GnRH antagonist protocol group. Furthermore, we did not find that these pre-defined factors affect other outcomes (**Supplementary Tables 5**, **6**).

### Publication bias

Funnel plots were not performed due to limited studies (<10).

## Discussion

### Summary of main findings

In this systematic review, we analyzed the efficacy of PPOS compared to conventional GnRH analogue protocols in women with PCOS undergoing IVF/ICSI. Our analysis found no evidence to support that the PPOS protocol could reduce the risk of OHSS, or increase the number of MII oocytes or live birth rates compared to the GnRH analogue protocols, either in RCTs or in observational studies. Additionally, the PPOS protocol required a higher dose of Gn and tended to improve the implantation rate (IR), clinical pregnancy rate (CPR), and ongoing pregnancy rate (OPR) in cohort studies. Moreover, the PPOS protocol had a higher IR, CPR, and OPR than the GnRH agonist short protocol, but no difference was found in these outcomes between the PPOS and the GnRH antagonist protocol. Furthermore, oral MPA or dydrogesterone required more doses of Gn than GnRH analogue protocols. Nevertheless, the certainty of evidence for the primary outcomes was low due to the imprecision and heterogeneity.

### Interpretation of results and clinical considerations

Patients with PCOS who undergo IVF/ICSI are at increased risk for OHSS, which is a potentially life-threatening complication of ovarian stimulation (35–37). Therefore, it is generally believed that the optimal management approach for infertility related to PCOS is to minimize the risk of OHSS while obtaining the best clinical outcomes during assisted reproductive technology. Our research found no significant difference in moderate or severe OHSS incidence between the PPOS and control groups, despite fewer events (2/741) in the PPOS group compared to the control group (10/714). Previous studies suggested that the progestin protocol stimulates the production of endogenous progestin (38, 39), which effectively inhibits luteinizing hormone (LH) and prevents OHSS, based on rat granulosa cells (8, 9). However, recent research on human granulosa cells showed that follicle-stimulating hormone (FSH) may increase the expression of 3B-HSD, leading to increased production of endogenous progestin without luteinization (40). Moreover, it is unclear whether the progestin produced during the stimulation process has any role or contribution to endogenous LH production. Therefore, further studies on mechanisms and studies with adequate sample sizes are needed to verify these findings.

In PCOS patients, hypersecretion of LH during the follicular phase can cause abnormal granulosa cell function (41), oocyte arrest or immaturity (42), and hinder the developmental potential of oocytes (41), resulting in decreased quality of oocytes and embryos (43). Our results indicate that PPOS achieves similar numbers of MII oocytes and good-quality embryos to GnRH analogue protocols without premature LH surges, indicating that the PPOS protocol effectively improves the quality of oocytes and embryos in PCOS patients. Several factors might explain this effectiveness. Administering progestin during the follicular phase can slow LH pulse frequency (44), block estrogen-induced LH surges (45), and promote oocyte health and cytoplasmic maturation (46). Additionally, the high proportion of progesterone to estrogen in the follicular fluid may lead to better embryo development (47).

Although there was a trend towards increased implantation rate, clinical pregnancy rate, and ongoing pregnancy rate, PPOS did not improve the live birth rate in patients with PCOS compared to GnRH analogue protocols. This inconsistency could be related to different control groups’ COS protocols. Subgroup analyses suggested comparable clinical outcomes between PPOS and the GnRH antagonist protocol. However, higher IR, CPR, and OPR in PPOS were found when the control group used the GnRH-a protocol. Considering that the GnRH antagonist protocol is widely used in PCOS patients due to its significant advantages over the agonist protocol (48–50), we believe that PPOS can achieve similar clinical outcomes as the GnRH antagonist protocol in patients with PCOS.

Our results suggested that PPOS needed a higher total dose of Gn stimulation with PCOS than the GnRH analogue protocols. This was consistent with previous studies (9, 13, 51). The possible theory is that the high progesterone milieu during PPOS leads to deeper pituitary suppression (13, 51), which will make follicles less sensitive to gonadotropin stimulation (8, 51). We also found that different oral progestins do not affect the primary outcomes in patients with PCOS, which is confirmed by recent findings (51, 52). However, unlike the Utrogestan group, we observed a significant increase in the total dose of Gn in the MPA and dydrogesterone subgroups. This increase may be related to the different bioavailability of progestins in the human body. For example, dydrogesterone is a derivative of natural progestin and has high bioavailability, while Utrogestan is microparticle progesterone with lower bioavailability in the human body after oral administration (53–55). As a result, different types of oral progestins may lead to varying degrees of pituitary suppression in COS cycles and differences in the total dose of Gn stimulation.

In addition to efficacy, we should not ignore the safety and cost of the COS protocols for patients with PCOS-related infertility. It is reasonable to suspect that long-term exposure to high levels of progestins might affect oocytes and embryos as well as fetal development. However, recent large sample size studies have shown no significant difference in the blastocyst euploidy rate (51, 56), neonatal outcomes (57, 58), or the risk of congenital malformations (57, 58) between PPOS and conventional GnRH analogue protocols. Even so, these studies cannot indicate the long-term safety of PPOS for children due to the lack of relevant data. Another potential issue hindering the PPOS protocol’s application is its cost. Evans et al. found that, compared to conventional GnRH analogue protocols for fresh embryo transplantation, the PPOS protocol resulted in a significantly higher cost per live birth, costing approximately an additional $10,000 and $5,000 compared to short agonist and antagonist protocols, respectively (59). However, the PPOS protocol is actually more cost-effective than other COS protocols for patients requiring the “freezing-all” strategy (33, 51), indicating the extra cost is mainly from embryo freezing and subsequent frozen-thawed embryo transfer (FET) (51). What’s more, it is noted that PCOS patients undergoing FET have a lower risk of OHSS and a higher LBR than fresh cycles (15, 60–62). Thus, given the high risk of OHSS and the potential benefit of FET for PCOS patients, the choice between the protocols may depend more on the patient’s condition and preference. For example, PPOS may be a better option if the patient plans to use a freezing strategy, like in preimplantation genetic testing or fertility preservation cycles.

### Strengths and limitations

Our study has its unique advantages. To our knowledge, this is the first meta-analysis that compared the efficacy of PPOS and GnRH analogue protocols on PCOS-related infertility until now. Unlike previous studies that mainly focused on the quality of oocytes and embryos, we also paid attention to clinical outcomes, such as the live birth rate. In addition, since our study was registered in INPLASY and adhered strictly to the Cochrane Handbook, all procedures were carried out faithfully. Moreover, we conducted subgroup analyses to identify possible factors that affect the outcomes, making our analysis more comprehensive.

The main limitations of this study are the different study designs of the included studies, including RCTs and cohort studies, which may introduce potential biases. To validate our findings, more high-quality RCTs are necessary. In addition, due to limited data from the included studies, we could not analyze and summarize newborn-related outcomes and the incidence of congenital disabilities. Furthermore, most of the included studies were conducted in China, and further verification is needed to determine consistency among different races and populations. Finally, the certainty of the evidence was generally low, mainly due to the precision of the estimates and substantial heterogeneity.

### Implication for future research

As PPOS is a new COS protocol that has emerged recently, few studies have explored its efficacy and safety in patients with PCOS-related infertility. The included studies are small sample sizes and are mainly conducted in China, so future large-scale, multi-ethnic studies are still needed. Several ongoing RCTs, such as NCT04175990 and NCT05112692, which plan to enroll more patients and explore neonatal outcomes, are expected to provide more evidence. In addition, future studies should pay more attention to the cost-effective analysis of the protocol for PCOS patients, which is essential for doctors and patients to make decisions. Furthermore, it would also be helpful to explore whether other potential factors, such as the administration mode of progestin, body mass index, and basal LH levels of patients, affect outcomes.

## Conclusions

In summary, there is no evidence to support that PPOS reduces the risk of OHSS or improves pregnancy outcomes in PCOS patients undergoing IVF/ICSI compared to GnRH analogue protocols. Still, the protocol may be a viable alternative, especially for frozen-thawed embryo transfer, due to its efficiency and safety. However, future randomized trials should consider the long-term safety of children and cost-effectiveness analyses.

## Data availability statement

The original contributions presented in the study are included in the article/**Supplementary Material**. Further inquiries can be directed to the corresponding author.

## Author contributions

LYang conceived the study and performed the study design. YY, LYao, and XL took part in the study selection. FL, QW, XL, and XZ participated in the data extraction and quality assessment. All authors contributed to the interpretation of the results. LYang conducted the statistical analysis and drafted the paper. All authors contributed to the article and approved the submitted version.
