# Supplementary material for: Efficacy of progestin-primed ovarian stimulation in women with polycystic ovary syndrome undergoing in vitro fertilization: a systematic review and meta-analysis
Source: Front Endocrinol (Lausanne). 2023 Sep 19;14:1224858. doi: 10.3389/fendo.2023.1224858 (PMC10546405; doi:10.3389/fendo.2023.1224858)
Supplement: Supplementary file 1 [file DataSheet_1.docx]

***Supplementary Material***

**Efficacy of progestin-primed ovarian stimulation in women with polycystic ovary syndrome undergoing in vitro fertilization:**

**A systematic review and meta-analysis**

**Liu Yang, Fuxiang Liang, Yue Yuan, Xufei Luo, Qi Wang, Liang Yao and Xuehong Zhang^*^**

*** Correspondence**: Corresponding Author: Xuehong Zhang

[zhangxueh@lzu.edu.cn](mailto:zhangxueh@lzu.edu.cn)

**1 Supplementary Figures**

**
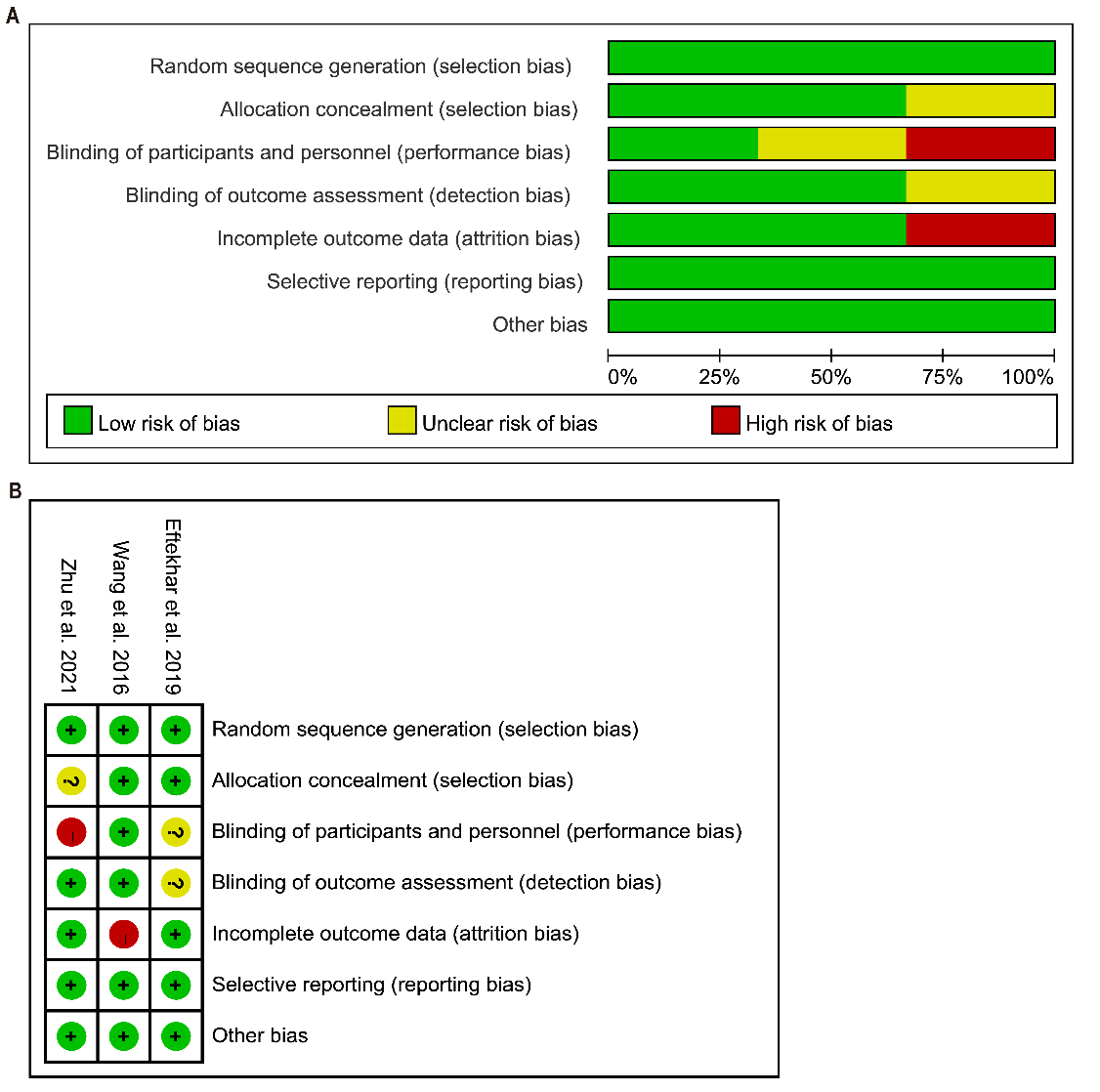
**

**Supplementary Figure 1.** Risk of bias assessment of included randomized controlled trials. **(A)** Risk of bias graph. **(B)** Risk of bias summary.


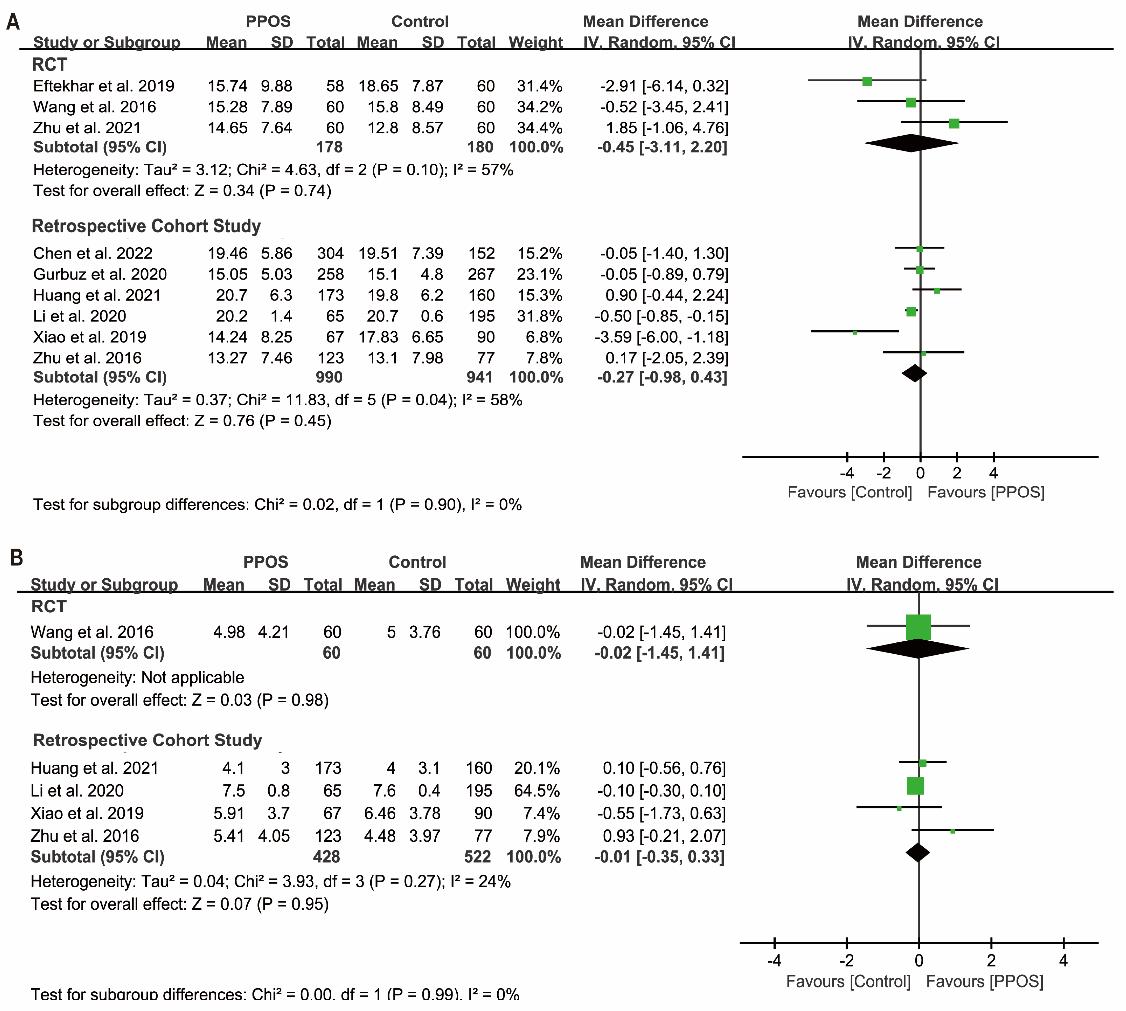


**Supplementary Figure 2.** Forest plots of the number of oocytes retrieved **(A)** and the number of good-quality embryos **(B)** in infertile patients with PCOS. Progestin-primed ovarian stimulation (PPOS) versus gonadotropin-releasing hormone (GnRH) analogue protocols.


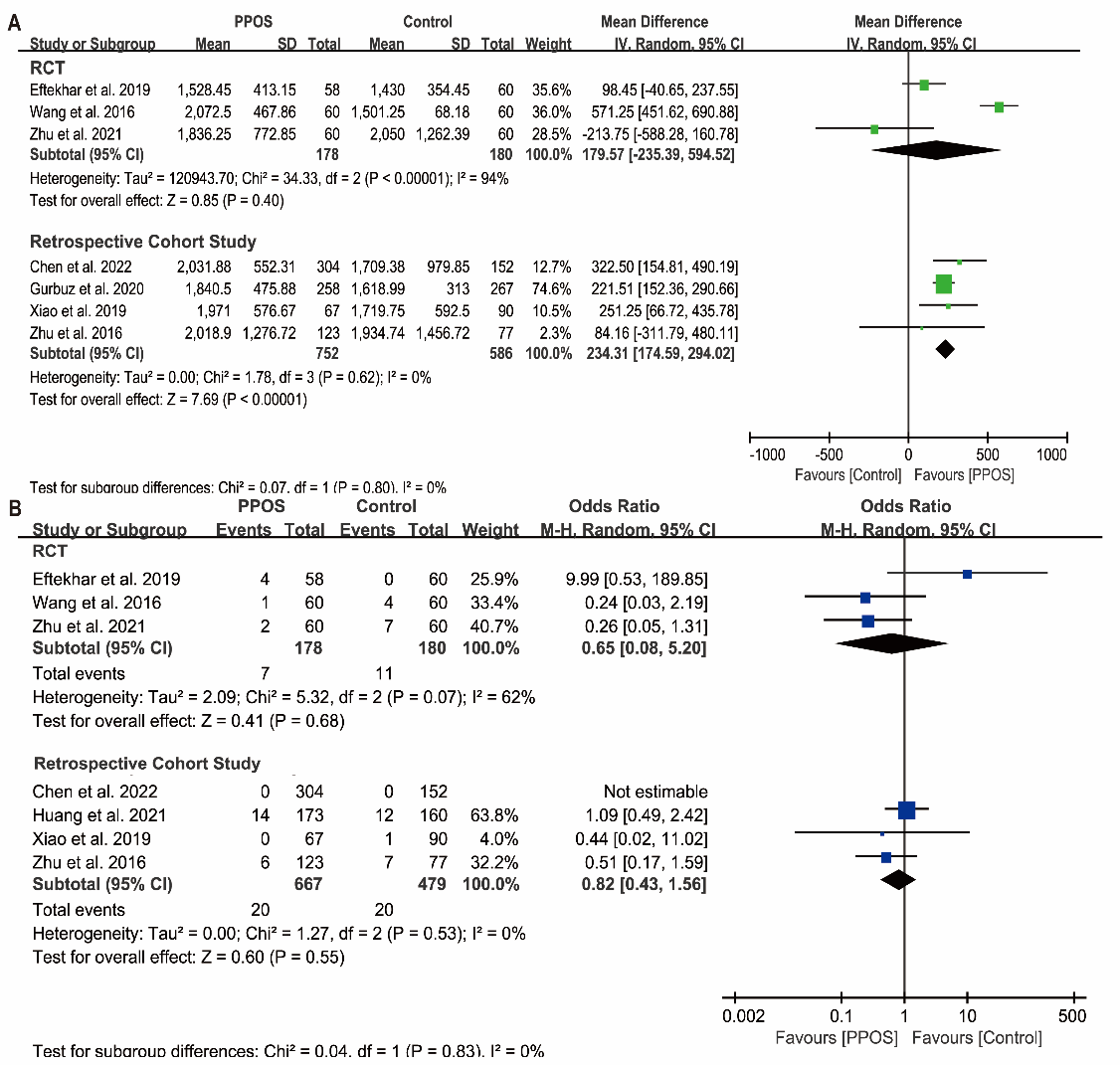


**Supplementary Figure 3.** Forest plots for total dose of gonadotropin (Gn) stimulation **(A)** and cycle cancellation rate **(B)** in infertile patients with PCOS. Progestin-primed ovarian stimulation (PPOS) versus gonadotropin-releasing hormone (GnRH) analogue protocols.


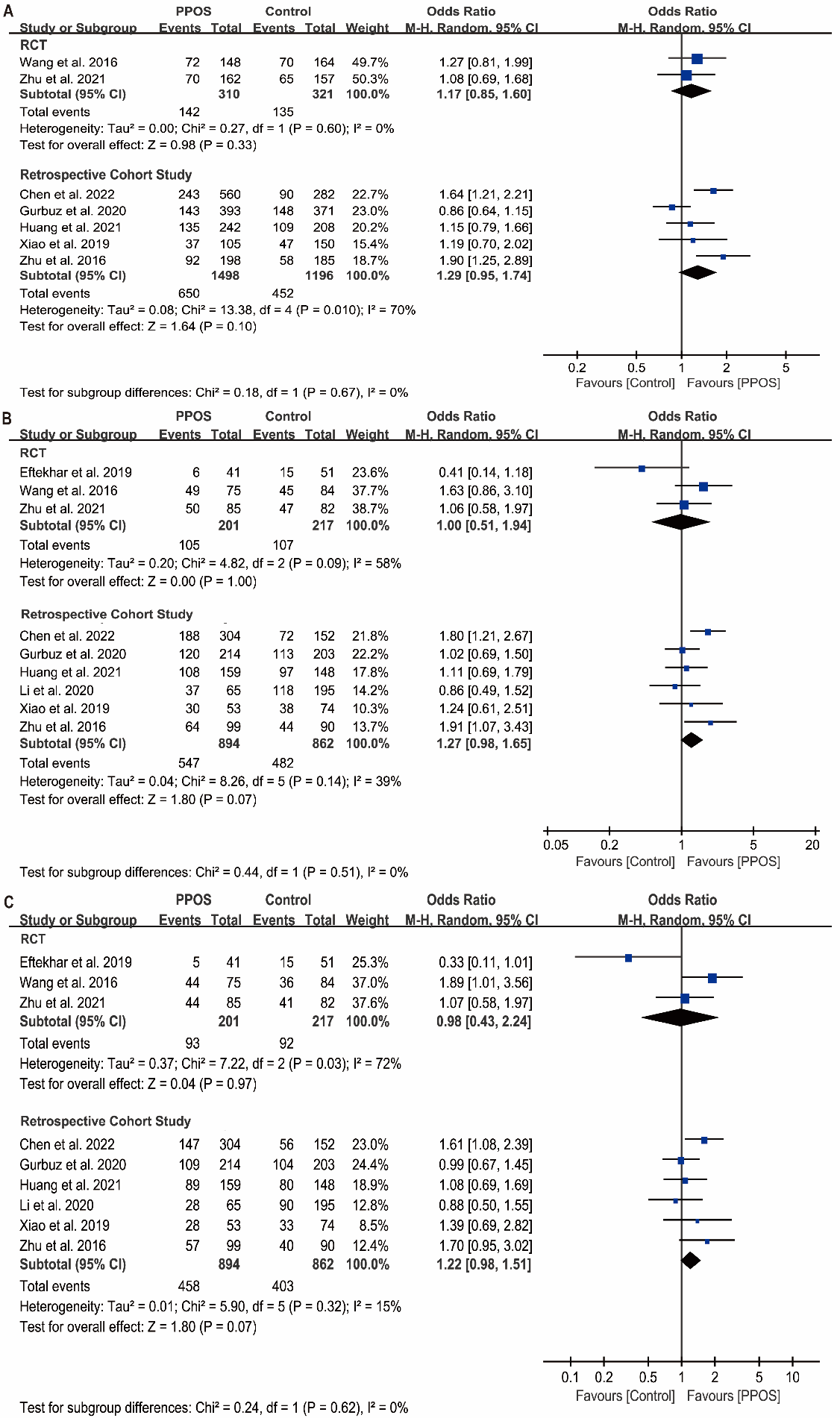


**Supplementary Figure 4.** Forest plots for implantation rate **(A)**, clinical pregnancy rate **(B)**, and ongoing pregnancy rate **(C)** in infertile patients with PCOS. Progestin-primed ovarian stimulation (PPOS) versus gonadotropin-releasing hormone (GnRH) analogue protocols.

**2 Supplementary Tables**

**Supplementary Table 1** PRISMA 2020 checklist.

| **Section and Topic** | **Item #** | **Checklist item** | **Location where item is reported** |
| --- | --- | --- | --- |
| **TITLE** | | |  |
| Title | 1 | Identify the report as a systematic review. | Page 1 |
| **ABSTRACT** | | |  |
| Abstract | 2 | See the PRISMA 2020 for Abstracts checklist. | Page 2 |
| **INTRODUCTION** | | |  |
| Rationale | 3 | Describe the rationale for the review in the context of existing knowledge. | Paras 1, 2, 3 |
| Objectives | 4 | Provide an explicit statement of the objective(s) or question(s) the review addresses. | Para 4 |
| **METHODS** | | |  |
| Eligibility criteria | 5 | Specify the inclusion and exclusion criteria for the review and how studies were grouped for the syntheses. | Para 4 |
| Information sources | 6 | Specify all databases, registers, websites, organisations, reference lists and other sources searched or consulted to identify studies. Specify the date when each source was last searched or consulted. | Para 3 |
| Search strategy | 7 | Present the full search strategies for all databases, registers and websites, including any filters and limits used. | Supplementary Table 1 |
| Selection process | 8 | Specify the methods used to decide whether a study met the inclusion criteria of the review, including how many reviewers screened each record and each report retrieved, whether they worked independently, and if applicable, details of automation tools used in the process. | Para 4 |
| Data collection process | 9 | Specify the methods used to collect data from reports, including how many reviewers collected data from each report, whether they worked independently, any processes for obtaining or confirming data from study investigators, and if applicable, details of automation tools used in the process. | Para 5 |
| Data items | 10a | List and define all outcomes for which data were sought. Specify whether all results that were compatible with each outcome domain in each study were sought (e.g. for all measures, time points, analyses), and if not, the methods used to decide which results to collect. | Para 4 |
|  | 10b | List and define all other variables for which data were sought (e.g. participant and intervention characteristics, funding sources). Describe any assumptions made about any missing or unclear information. | Para 4 |
| Study risk of bias assessment | 11 | Specify the methods used to assess risk of bias in the included studies, including details of the tool(s) used, how many reviewers assessed each study and whether they worked independently, and if applicable, details of automation tools used in the process. | Para 7 |
| Effect measures | 12 | Specify for each outcome the effect measure(s) (e.g. risk ratio, mean difference) used in the synthesis or presentation of results. | Para 9 |
| Synthesis methods | 13a | Describe the processes used to decide which studies were eligible for each synthesis (e.g. tabulating the study intervention characteristics and comparing against the planned groups for each synthesis (item #5)). | Para 9 |
|  | 13b | Describe any methods required to prepare the data for presentation or synthesis, such as handling of missing summary statistics, or data conversions. | Para 9 |
|  | 13c | Describe any methods used to tabulate or visually display results of individual studies and syntheses. | Para 9 |
|  | 13d | Describe any methods used to synthesize results and provide a rationale for the choice(s). If meta-analysis was performed, describe the model(s), method(s) to identify the presence and extent of statistical heterogeneity, and software package(s) used. | Para 10 |
|  | 13e | Describe any methods used to explore possible causes of heterogeneity among study results (e.g. subgroup analysis, meta-regression). | Para 10 |
|  | 13f | Describe any sensitivity analyses conducted to assess robustness of the synthesized results. | Not applicable |
| Reporting bias assessment | 14 | Describe any methods used to assess risk of bias due to missing results in a synthesis (arising from reporting biases). | Para 7 |
| Certainty assessment | 15 | Describe any methods used to assess certainty (or confidence) in the body of evidence for an outcome. | Para 8 |
| **RESULTS** | | |  |
| Study selection | 16a | Describe the results of the search and selection process, from the number of records identified in the search to the number of studies included in the review, ideally using a flow diagram. | Para 1, Figure 1 |
|  | 16b | Cite studies that might appear to meet the inclusion criteria, but which were excluded, and explain why they were excluded. | Para 1 |
| Study characteristics | 17 | Cite each included study and present its characteristics. | Para 2, Table 1 |
| Risk of bias in studies | 18 | Present assessments of risk of bias for each included study. | Para 3, Supplementary Figure 1, Supplementary Table 2 |
| Results of individual studies | 19 | For all outcomes, present, for each study: (a) summary statistics for each group (where appropriate) and (b) an effect estimate and its precision (e.g. confidence/credible interval), ideally using structured tables or plots. | Figure 2, Supplementary Figure 2, Supplementary Figure 3, Supplementary Figure 4, Text |
| Results of syntheses | 20a | For each synthesis, briefly summarise the characteristics and risk of bias among contributing studies. | Supplementary Figure 1, Text |
|  | 20b | Present results of all statistical syntheses conducted. If meta-analysis was done, present for each the summary estimate and its precision (e.g. confidence/credible interval) and measures of statistical heterogeneity. If comparing groups, describe the direction of the effect. | Figure 2, Supplementary Figure 2, Supplementary Figure 3, Supplementary Figure 4, Text |
|  | 20c | Present results of all investigations of possible causes of heterogeneity among study results. | Supplementary Table 4, Supplementary Table 5 |
|  | 20d | Present results of all sensitivity analyses conducted to assess the robustness of the synthesized results. | Not applicable |
| Reporting biases | 21 | Present assessments of risk of bias due to missing results (arising from reporting biases) for each synthesis assessed. | Supplementary Figure 1 |
| Certainty of evidence | 22 | Present assessments of certainty (or confidence) in the body of evidence for each outcome assessed. | Table 2, Supplementary Table 3 |
| **DISCUSSION** | | |  |
| Discussion | 23a | Provide a general interpretation of the results in the context of other evidence. | Para 1 |
|  | 23b | Discuss any limitations of the evidence included in the review. | Para 8 |
|  | 23c | Discuss any limitations of the review processes used. | Para 8 |
|  | 23d | Discuss implications of the results for practice, policy, and future research. | Para 9 |
| **OTHER INFORMATION** | | |  |
| Registration and protocol | 24a | Provide registration information for the review, including register name and registration number, or state that the review was not registered. | Para 1 |
|  | 24b | Indicate where the review protocol can be accessed, or state that a protocol was not prepared. | Para 1 |
|  | 24c | Describe and explain any amendments to information provided at registration or in the protocol. | Para 1 |
| Support | 25 | Describe sources of financial or non-financial support for the review, and the role of the funders or sponsors in the review. | Funding section |
| Competing interests | 26 | Declare any competing interests of review authors. | Conflict of interest section |
| Availability of data, code and other materials | 27 | Report which of the following are publicly available and where they can be found: template data collection forms; data extracted from included studies; data used for all analyses; analytic code; any other materials used in the review. | Not applicable |

**Supplementary Table 2** Search strategy for PubMed (from inception to April 1, 2023).

| **Search** |  | **Results** |
| --- | --- | --- |
| #1 | ("polycystic ovarian syndrome"[Title/Abstract] OR ("ovary syndrome polycystic"[Title/Abstract] OR "syndrome polycystic ovary"[Title/Abstract] OR "stein leventhal syndrome"[Title/Abstract] OR "stein leventhal syndrome"[Title/Abstract] OR "syndrome stein leventhal"[Title/Abstract] OR "sclerocystic ovarian degeneration"[Title/Abstract] OR ((("Ovarian"[All Fields] OR "ovarians"[All Fields]) AND ("degenerate"[All Fields] OR "degenerated"[All Fields] OR "degenerately"[All Fields] OR "degenerates"[All Fields] OR "degenerating"[All Fields] OR "Degeneration"[All Fields] OR "degenerations"[All Fields])) AND "Sclerocystic"[Title/Abstract]) OR "sclerocystic ovary syndrome"[Title/Abstract] OR "polycystic ovarian syndrome"[Title/Abstract] OR "ovarian syndrome polycystic"[Title/Abstract] OR "polycystic ovary syndrome 1"[Title/Abstract] OR "sclerocystic ovaries"[Title/Abstract] OR (("ovarial"[All Fields] OR "Ovary"[MeSH Terms] OR "Ovary"[All Fields] OR "Ovaries"[All Fields] OR "ovary s"[All Fields]) AND "Sclerocystic"[Title/Abstract]) OR "sclerocystic ovary"[Title/Abstract]) OR "Polycystic Ovary Syndrome"[MeSH Terms]) | 19177 |
| #2 | ("progestin-primed ovarian stimulation"[Title/Abstract] OR "progest*"[Title/Abstract] OR "Gestagen"[Title/Abstract] OR "dydrogesterone*"[Title/Abstract] OR "duphaston"[Title/Abstract] OR "gestrinone*"[Title/Abstract] OR "utrogestan"[Title/Abstract] OR "medroxyprogesterone*"[Title/Abstract] OR "medrogestone"[Title/Abstract] OR "depoprovera"[Title/Abstract] OR "depot medroxyprogesterone"[Title/Abstract] OR "Progestins"[MeSH Terms] OR "Progesterone"[MeSH Terms] OR "Desogestrel"[MeSH Terms] OR "Dydrogesterone"[MeSH Terms]) | 139960 |
| #3 | #1 AND # 2 | 1720 |

**Supplementary Table 3** Newcastle-Ottawa Quality Assessment Scale of included observational studies.

| Study | Design | Selection | | | | Comparability | Outcome | | | Summary score (risk of bias) | Final quality assessment |
| --- | --- | --- | --- | --- | --- | --- | --- | --- | --- | --- | --- |
|  |  | Representative-ness of the exposed cohort | Selection of non-exposed cohort | Ascertainment of exposure | Demonstration that the outcome of interest was not present at the start of the study | Comparability of cohorts on the basis of the design or analysis | Assessment of outcome | Was follow-up long enough for outcomes to occur | Adequacy of follow-up of cohorts |  |  |
| Xiao et al. | Retrospective  cohort study | 🟊 | 🟊 | 🟊 | ☆ | 🟊 | 🟊 | 🟊 | ☆ | 6 | Unclear risk |
| Zhu et al. | Retrospective  cohort study | 🟊 | 🟊 | 🟊 | ☆ | 🟊 | 🟊 | 🟊 | ☆ | 6 | Unclear risk |
| Gurbuz et al. | Retrospective  cohort study | 🟊 | 🟊 | 🟊 | ☆ | 🟊 | 🟊 | 🟊 | ☆ | 6 | Unclear risk |
| Huang et al. | Retrospective  cohort study | 🟊 | 🟊 | 🟊 | ☆ | 🟊 | 🟊 | 🟊 | 🟊 | 7 | Low risk |
| Chen et al. | Retrospective  cohort study | 🟊 | 🟊 | 🟊 | ☆ | 🟊 | 🟊 | 🟊 | 🟊 | 7 | Low risk |
| Li et al. | Retrospective  cohort study | 🟊 | 🟊 | 🟊 | ☆ | 🟊 | 🟊 | 🟊 | ☆ | 6 | Unclear risk |

**Supplementary Table 4** The certainty of the evidence for secondary outcomes.

| **Outcomes** | **Study design** | **Anticipated absolute effects* (95% CI)** | | | **Relative effect**  **(95% CI)** | **No. Of cycles**  **(studies)** | **Quality of**  **the evidence**  **(GRADE)** |
| --- | --- | --- | --- | --- | --- | --- | --- |
|  |  | **Risk with control** | **Risk with PPOS** | **Difference** |  |  |  |
| **Cycle cancellation** | **RCT** | 61 per 1000 | 40 per 1000  (5 to 317) | -21 (-56 to 256) | **OR** 0.65  (0.08 to 5.20) | 358 patients  (3) | **Low^1^** |
|  | **Cohort** | 42 per 1000 | 34 per 1000  (18 to 66) | -8 (-24 to 24) | **OR** 0.82  (0.43 to 1.56) | 1146 patients  (4) | **Low** |
| **Implantation** | **RCT** | 421 per 1000 | 493 per 1000  (358 to 674) | 72 (-63 to 253) | **OR** 1.17  (0.85 to 1.60) | 631 embryos  (2) | **Moderate^2^** |
|  | **Cohort** | 378 per 1000 | 488 per 1000  (359 to 658) | 110 (-19 to 280) | **OR** 1.29  (0.95 to 1.74) | 2694 embryos  (5) | **Low** |
| **Clinical pregnancy** | **RCT** | 493 per 1000 | 493 per 1000  (251 to 956) | 0 (-242 to 463) | **OR** 1.00  (0.51 to 1.94) | 418(3) | **Low^1^** |
|  | **Cohort** | 559 per 1000 | 710 per 1000  (548 to 922) | 151 (-11 to 363) | **OR** 1.27  (0.98 to 1.65) | 1756 (6) | **Low** |
| **Ongoing pregnancy** | **RCT** | 424 per 1000 | 416 per 1000  (182 to 950) | -8 (-242 to 526) | **OR** 0.98  (0.43 to 2.24) | 418(3) | **Low^1^** |
|  | **Cohort** | 468 per 1000 | 571 per 1000  (459 to 707) | 103 (-9 to 239) | **OR** 1.22  (0.98 to 1.51) | 1756 (6) | **Low** |
| **Total dose of Gn stimulation** | **RCT** | The mean total dose of Gn stimulation in the control group was 1660.42 IU. | The mean total dose of Gn stimulation in the intervention group was 179.57 IU higher (235.39 lower to 594.52 higher). | 179.57 (-235.39 to 594.52) | NA | 358 (3) | **Low^1^** |
|  | **Cohort** | The mean total dose of Gn stimulation in the control group was 1755.18 IU. | The mean total dose of Gn stimulation in the intervention group was 234.31 IU higher (174.59 higher to 294.02 higher). | 234.31 (174.59 to 294.02) | NA | 1338 (4) | **Low** |
| **Oocytes retrieved** | **RCT** | The mean number of oocytes retrieved in the control group was 15.75. | The mean number of oocytes retrieved in the intervention group was 0.45 lower (3.11 lower to 2.20 higher). | -0.45 (-3.11 to 2.20) | NA | 358 patients  (3) | **Low^1^** |
|  | **Cohort** | The mean number of oocytes retrieved in the control group was 17.87. | The mean number of oocytes retrieved in the intervention group was 0.27 lower (0.98 lower to 0.43 higher). | -0.27 (-0.98 to 0.43) | NA | 1931 patients  (6) | **Low** |
| **Good-quality embryos** | **RCT** | The mean number of good-quality embryos in the control group was 5. | The mean number of good-quality embryos in the intervention group was 0.02 lower (1.45 lower to 1.41 higher). | -0.02 (-1.45 to 1.41) | NA | 120 patients  (1) | **Low^3^** |
|  | **Cohort** | The mean number of good-quality embryos in the control group was 5.84. | The mean number of good-quality embryos in the intervention group was 0.01 lower (0.35 lower to 0.33 higher). | -0.01 (-0.35 to 0.33) | NA | 950 patients  (4) | **Low** |

*The risk in the intervention group (and the 95% confidence interval) is based on the risk in the control group and the relative effect of the intervention.

CI, confidence interval; Gn, gonadotropin; GRADE, Grading of Recommendation, Assessment, Development, and Evaluation; NA, not available; OR, odds ratio; PPOS, progestin-primed ovarian stimulation; RCT, randomized controlled trial.

^1^Downgraded two steps due to imprecision of results as shown in wide confidence intervals and heterogeneity.

^2^Downgraded one step due to imprecision of results as shown in wide confidence intervals.

^3^Downgraded two steps due to wide confidence intervals.

**Supplementary Table 5** Subgroup analyses of primary outcomes.

| **Subgroups** | **No. of studies** | **Statistical method**  **(Random-effects-model)** | **Effect estimate**  **(95% CI)** | **Heterogeneity**  **(%)** | **Test for**  **subgroup**  **differences (P value)** |
| --- | --- | --- | --- | --- | --- |
| **Live birth rate per transfer (%)** | | | | | |
| Total | 5 |  | 1.01 [0.66, 1.55] | 70 |  |
| GnRH antagonist protocol | 3 | Odds ratio | 1.08 [0.80, 1.45] | 0 | 0.73 |
| Short protocol | 2 |  | 0.85 [0.23, 3.10] | 92 |  |
| MPA | 2 | Odds ratio | 1.23 [0.69, 2.21] | 65 | 0.79 |
| Dydrogesterone | 1 |  | 1.04 [0.68, 1.60] | NA |  |
| Utrogestan | 2 |  | 0.79 [0.24, 2.64] | 86 |  |
| **Incidence of OHSS (%)** | | | | | |
| Total | 6 |  | 0.30 [0.07, 1.27] | 0 |  |
| GnRH antagonist protocol | 4 | Odds ratio | 0.10 [0.01, 1.74] | NA | 0.53 |
| Short protocol | 2 |  | 0.44 [0.08, 2.32] | 0 |  |
| MPA | 2 | Odds ratio | 0.13 [0.02, 1.10] | 0 | 0.57 |
| Dydrogesterone | 2 |  | NA | NA |  |
| Utrogestan | 2 |  | 0.62 [0.09, 4.49] | NA |  |
| **No. of MII oocytes** | | | | | |
| Total | 6 |  | -0.01 [-0.89, 0.87] | 57 |  |
| GnRH antagonist protocol | 4 | Mean difference | -0.00 [-1.17, 1.16] | 73 | 0.79 |
| Short protocol | 2 |  | -0.26 [-1.79, 1.26] | 0 |  |
| MPA | 1 | Mean difference | -0.33 [-2.78, 2.12] | NA | 0.88 |
| Dydrogesterone | 3 |  | -0.22 [-1.55, 1.11] | 82 |  |
| Utrogestan | 2 |  | 0.25 [-1.31, 1.81] | 0 |  |

CI, confidence interval; GnRH, gonadotropin-releasing hormone; MII, metaphase two; MPA, medroxyprogesterone acetate; NA, not available; OHSS, ovarian hyperstimulation syndrome.

*The difference between subgroups was statistically significant.

**Supplementary Table 6** Subgroup analyses of secondary outcomes.

| **Subgroups** | **No. of studies** | **Statistical method**  **(Random-effects**  **-model)** | **Effect estimate (95% CI)** | **Heterogeneity (%)** | **Test for**  **subgroup**  **differences (P value)** |
| --- | --- | --- | --- | --- | --- |
| **The total dose of Gn stimulation (IU)** | | | | | |
| Total | 7 |  | 231.14 [78.61, 383.68] | 85 |  |
| GnRH antagonist protocol | 4 | Mean difference | 155.67 [34.76, 276.57] | 59 | 0.11 |
| Short protocol | 3 |  | 375.76 [131.14, 620.37] | 79 |  |
| MPA | 3 | Mean difference | 390.77 [184.34, 597.20] | 81 | 0.03* |
| Dydrogesterone | 2 |  | 175.39 [58.63, 292.14] | 59 |  |
| Utrogestan | 2 |  | -72.00 [-363.61, 219.60] | 13 |  |
| **No. of oocytes retrieved** | | | | | |
| Total | 9 |  | -0.29 [-0.97, 0.39] | 51 |  |
| GnRH antagonist protocol | 6 | Mean difference | -0.41 [-1.35, 0.53] | 69 | 0.63 |
| Short protocol | 3 |  | -0.06 [-1.13, 1.01] | 0 |  |
| MPA | 4 | Mean difference | -0.82 [-1.91, 0.26] | 55 | 0.29 |
| Dydrogesterone | 3 |  | -0.08 [-1.42, 1.27] | 58 |  |
| Utrogestan | 2 |  | 0.79 [-0.97, 2.55] | 0 |  |
| **No. of good-quality embryos** | | | | | |
| Total | 5 |  | -0.07 [-0.25, 0.12] | 0 |  |
| GnRH antagonist protocol | 3 | Mean difference | -0.09 [-0.29, 0.10] | 0 | 0.17 |
| Short protocol | 2 |  | 0.56 [-0.35, 1.47] | 4 |  |
| MPA | 3 | Mean difference | -0.11 [-0.31, 0.09] | 0 | 0.18 |
| Dydrogesterone | 1 |  | 0.10 [-0.56, 0.76] | NA |  |
| Utrogestan | 1 |  | 0.93 [-0.21, 2.07] | NA |  |
| **Cycle cancellation rate (%)** | | | | | |
| Total | 7 |  | 0.66 [0.31, 1.43] | 29 |  |
| GnRH antagonist protocol | 4 | Odds ratio | 0.87 [0.26, 2.87] | 43 | 0.39 |
| Short protocol | 3 |  | 0.44 [0.16, 1.20] | 0 |  |
| MPA | 3 | Odds ratio | 0.29 [0.05, 1.80] | 0 | 0.30 |
| Dydrogesterone | 2 |  | 2.10 [0.28, 15.99] | 53 |  |
| Utrogestan | 2 |  | 0.41 [0.16, 1.04] | 0 |  |
| **Clinical pregnancy rate per transfer (%)** | | | | | |
| Total | 9 |  | 1.22 [0.96, 1.55] | 41 |  |
| GnRH antagonist protocol | 6 | Odds ratio | 1.00 [0.80, 1.25] | 0 | 0.002* |
| Short protocol | 3 |  | 1.79 [1.34, 2.40] | 0 |  |
| MPA | 4 | Odds ratio | 1.38 [0.97, 1.95] | 36 | 0.29 |
| Dydrogesterone | 3 |  | 0.95 [0.65, 1.39] | 31 |  |
| Utrogestan | 2 |  | 1.44 [0.81, 2.55] | 46 |  |
| **Ongoing pregnancy rate per transfer (%)** | | | | | |
| Total | 9 |  | 1.20 [0.95, 1.51] | 39 |  |
| GnRH antagonist protocol | 6 | Odds ratio | 0.74 [0.46, 1.20] | 76 | 0.02* |
| Short protocol | 3 |  | 1.51 [1.06, 2.14] | 0 |  |
| MPA | 4 | Odds ratio | 0.88 [0.38, 2.07] | 87 | 0.37 |
| Dydrogesterone | 3 |  | 0.89 [0.58, 1.38] | 47 |  |
| Utrogestan | 2 |  | 1.36 [0.87, 2.13] | 13 |  |
| **Implantation rate (%)** | | | | | |
| Total | 7 |  | 1.25 [1.00, 1.56] | 57 |  |
| GnRH antagonist protocol | 4 | Odds ratio | 1.01 [0.83, 1.22] | 0 | 0.001* |
| Short protocol | 3 |  | 1.61 [1.30, 1.99] | 0 |  |
| MPA | 3 | Odds ratio | 1.45 [1.16, 1.81] | 0 | 0.08 |
| Dydrogesterone | 2 |  | 0.97 [0.74, 1.28] | 28 |  |
| Utrogestan | 2 |  | 1.44 [0.82, 2.51] | 70 |  |

CI, confidence interval; Gn, gonadotropin; GnRH, gonadotropin-releasing hormone; MPA, medroxyprogesterone acetate; NA, not available.

*The difference between subgroups was statistically significant.
